# Alleviation of Surgery-Induced Osteitis in Sinonasal Cavity by Dexamethasone-Loaded Poly(lactic-*co*-glycolic acid) (PLGA) Microparticles with Strong Calcium-Binding Affinity

**DOI:** 10.3390/pharmaceutics14030546

**Published:** 2022-02-28

**Authors:** Seung-No Hong, Minjae Kim, Jin-A Park, Minji Kang, Hyunkyung Cha, Sohyun Park, Joon Kon Kim, Jinyoung Pac, Yuju Seo, Sungwhan Kim, Minju Kim, Dae Woo Kim, Yan Lee

**Affiliations:** 1 Department of Otolaryngology-Head and Neck Surgery, Seoul National University Boramae Medical Center, 5 Gil 20, Boramae-ro, Dongjak-gu, Seoul 07061, Korea; seuno77@naver.com (S.-N.H.); jina2604@hanmail.net (J.-A.P.); hkcha26@gmail.com (H.C.); auminju@gmail.com (M.K.); 2 Sensory Organ Research Institute, Seoul National University Medical Research Center, Seoul 03080, Korea; 3 Department of Chemistry, Seoul National University, Gwanak-ro 1, Gwanak-gu, Seoul 08826, Korea; mjkim46@snu.ac.kr (M.K.); applekang@snu.ac.kr (M.K.); ssohyun@snu.ac.kr (S.P.); jypac@snu.ac.kr (J.P.); king9410@snu.ac.kr (S.K.); 4 Department of Otolaryngology-Head and Neck Surgery, Daejeon Eulji Medical Center, Eulji University, Daejeon 35233, Korea; jooonn@hanmail.net; 5 Department of Otolaryngology-Head and Neck Surgery, Chungnam National University Hospital, Chungnam National University, Daejeon 35015, Korea; yuz1121@naver.com

**Keywords:** chronic rhinosinusitis, osteitis, dexamethasone, sinonasal cavity, calcium binding, poly(lactic-*co*-glycolic acid) (PLGA)

## Abstract

For the treatment of sinus surgery-induced osteitis in chronic rhinosinusitis (CRS), oral or intranasal administration of corticoids is generally used, although it has critical limitations and unavoidable side effects. To overcome these limitations, we designed dexamethasone (Dex)-loaded poly(lactic-*co*-glycolic acid) (PLGA) microparticles with bone-specific binding affinity, which could release the encapsulated Dex in a sustained manner on the exposed bone after the surgical wound in the nasal cavity. In a previous report, we prepared poly(butyl methacrylate-*co*-methacryloyloxyethyl phosphate) (PBMP) with both calcium-binding phosphomonoester groups and PLGA-binding butyl groups to introduce strong calcium-binding property to PLGA particles. In this study, after successful encapsulation of Dex in the PBMP-coated PLGA particles, we applied the Dex-PLGA/PBMP to the treatment of post-operative osteitis in the sinonasal cavity. The Dex-PLGA/PBMP showed more than 5-times higher binding affinity to the hydroxyapatite (HA) surface compared to the non-coated PLGA particles, without altering the morphology and encapsulation efficiency. After establishing the neo-osteogenesis mouse model by mechanical injury of the nasal mucosa, the activity of intranasally administered Dex-PLGA/PBMP was examined to inhibit the formation of undesirable new woven bone during the wound healing process. In addition, significantly lower osteocalcin activity was observed in the group treated with Dex-PLGA/PBMP, indicating decreased activation of osteoblasts. Overall, these results demonstrate that the PLGA/PBMP microparticle strategy has great potential for the treatment of CRS-related osteitis by localized corticoid delivery on the exposed bones with minimal side effects.

## 1. Introduction

Chronic rhinosinusitis (CRS), characterized by heterogeneous inflammatory conditions of the nose and paranasal sinuses lasting for more than 12 weeks, is one of the most frequent chronic inflammatory diseases of the upper airway [1]. CRS is a major financial burden to the health care system of the upper airway, and CRS patients suffer from severe nasal congestion, as well as accompanying inflammation. The treatment of CRS includes modulation of inflammation in the nasal mucosa, facilitation of the secretion drainage, and, if possible, restoration of mucociliary clearance in the sinuses [2], but surgical treatment is required for patients with limited response to medical therapy.

Although the effectiveness of endoscopic sinus surgery (ESS) in treating CRS is well established [3,4], more than 50% of patients who had undergone surgery showed recurrence at 3 years post-surgery and required revision surgery [5]. Previous studies have identified that osteitis in the nasal bones is closely related to CRS recurrence [4,6]. Although the pathogenesis of CRS-related osteitis is still not clearly understood, several studies have suggested an association between osteitis and revision sinus surgery. There was a positive correlation between computed tomography (CT) scores of osteitis and the number of sinonasal operations [7,8]. In addition, osteitis of the ethmoid bone was more frequently observed in patients undergoing revision than in those undergoing primary surgery [9].

Patients with osteitis show poor prognostic outcomes after CRS surgery [10,11]. Osteitis could delay the wound healing process of surgical points to deteriorate the regeneration of the mucosal membrane, and it might become the focal point of inflammation to initiate the recurrence of CRS [12]. Therefore, it is expected that the effective cure of post-ESS osteitis could facilitate wound healing and alleviate the recurrence of CRS. 

To treat CRS-related osteitis, radical surgery to remove all involved bones and long-term treatment with antibiotics have been suggested with limited success [6]. Since corticosteroids are quite effective in reducing inflammatory cascades and promoting wound healing after surgery [2,13,14], intranasal or oral administration of corticoids might be one of the best available options for the treatment of CRS-related osteitis [2,12]. However, the systemic use of corticoids can provoke serious side effects [15]. Local intranasal usage of corticoids can be free from this issue, but the corticoids delivered by the nasal spray or nasal irrigation are rapidly diffused and washed out from the affected area, which requires frequent administration [2]. For the prolonged effect of corticoid activity after the ESS, various intranasal topical treatment methods, including microparticles [16] or hydrogels [17], were suggested. However, there is no previous report focused on the treatment of CRS-related osteitis. If corticoids can be maintained above the effective concentration only near the affected osteitic area for a longer duration, it is expected that the development of osteitis could be effectively inhibited to enhance wound healing after surgery as well as to prevent further recurrence of CRS by a single administration of corticoids with minimal side effects. 

**Figure 1:**
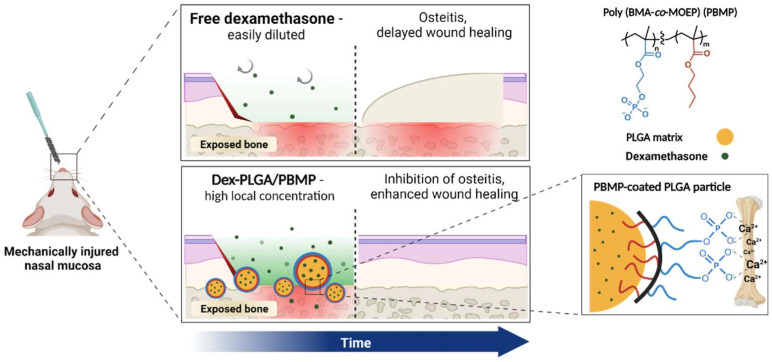
Schematic illustration of the Dex-loaded PLGA/PBMP microparticles on exposed bone surfaces in mouse nasal cavity for maintenance of the local Dex concentration around surgery-induced osteitis.

Herein, we report the effect of corticoid-releasing microparticles with specific bone-binding affinity to the osteitis of nasal bones and wound healing in a mouse model. In a previous report, we developed poly(lactic-*co*-glycolic acid) (PLGA) microparticles coated with a calcium-binding polymer, poly(butyl methacrylate-*co*-methacryloyloxyethyl phosphate) (PBMP) [16]. PLGA is an FDA-approved biodegradable polymer, and PLGA-based microparticles allow tunable release of encapsulated drugs [17,18]. PBMP contains both phosphomonoester groups for calcium binding and butyl groups for hydrophobic PLGA binding, respectively, and can be coated on the hydrophobic surface of PLGA microparticles to introduce an adhesive property on calcium-rich tissues. We selected dexamethasone (Dex) as a model corticoid drug, which can reduce the inflammatory sinonasal mucosa [19] and promote wound repair following the surgical procedure [20]. We intended to maintain the Dex concentration for a longer duration only near the exposed bones after the surgery through sustained Dex release from the PLGA/PBMP particles, which have a specific binding affinity to the calcium-rich tissues (Figure 1).

In this study, we show that Dex can be encapsulated into the PLGA microparticles by the oil-in-water single emulsion method and that the particles can obtain strong binding affinity to hydroxyapatite (HA), the main component of the bone, by the PBMP coating. The Dex-encapsulated PLGA/PBMP particles were intranasally administered by instillation to a mouse osteitis model, which was established by the mechanical injury of the mouse nasal mucosa. The anti-osteitic activity of the Dex-encapsulated PLGA/PBMP particles was compared to that of non-encapsulated particles and free Dex by quantification of woven bone formation and osteoblast activation in the wounded area of the mouse nasal tissue.

## 2. Materials and Methods

### 2.1. Materials

Poly(d,l-lactide-*co*-glycolide; average *M*_w_ = 54,000–69,000; lactide: glycolide = 50:50) was purchased from Evonik (Essen, Germany). Polyvinyl alcohol (PVA) (average *M*_w_ = 13,000–23,000), 2-hydroxyethyl methacrylate (HEMA), pyridine, n-butyl methacrylate (BMA), chloroform, 2,2′-azobis(2-methylpropionitrile) (AIBN), ethanol (EtOH), and tetrahydrofuran (THF) were purchased from Sigma Aldrich (St. Louis, MO, USA). Dimethyl chlorophosphate (DCP) was purchased from Acros Organics (Geel, Belgium). Dexamethasone (Dex) and bromotrimethylsilane (TMSBr) were purchased from TCI (Tokyo, Japan). Cyanine 5.5-amine (Cy5.5) was purchased from Lumiprobe (Hunt Valley, MD, USA). Dichloromethane (DCM), methanol (MeOH), dimethyl sulfoxide (DMSO) and acetonitrile (ACN) were purchased from Samchun (Seoul, Korea). Hydroxyapatite discs (9.5 mm diameter × 2 mm thick) were purchased from Clarkson Chromatography Products (South Williamsport, PA, USA).

### 2.2. Synthesis of PBMP

The dimethyl methacryloyloxyethyl phosphate (DMOEP) monomer and the PBMP polymer were synthesized according to a previously reported procedure [16,21]. First, the mixture of DCP (120 mmol) and pyridine (120 mmol) was slowly added to 250 mL of chloroform containing 100 mmol of HEMA on ice. The mixture was stirred for 2 h and washed with 0.01 M HCl, NaHCO_3_ and brine. The organic layer was collected and evaporated to obtain the DMOEP monomer as pale-yellow oil. 

Then, DMOEP (14 mmol) and BMA (14 mmol) were dissolved in the mixture of EtOH and THF (28 mL, *v*/*v* = 4:1). For the initiation of radical polymerization, AIBN (0.28 mmol) was added to the solution and the mixture was incubated with overnight stirring at 60 °C. After evaporization of the solvent, poly(BMA-*co*-DMOEP) was obtained as a transparent oil form. The polymer was demethylated by using TMSBr. The polymer in anhydrous chloroform (40 mL) was slowly added with TMSBr (56 mmol). The mixed solution was first stirred in an ice bath for 1 h and at ambient temperature for another 2 h. Following the evaporation of the solvent, 100 mL of H_2_O/THF with a volume ratio of 1:7 was added. After overnight reaction at ambient temperature, the solvent was evaporated. The resulting product was dialyzed against MeOH and deionized water using a dialysis membrane (MWCO = 3000 g/mol; Spectrum Labs, USA). After freeze drying, PBMP was obtained as a white powder with the purified yield of 68.5%.

### 2.3. Gel Permeation Chromatography (GPC)

GPC was used for analyzing the molecular weight distribution of PBMP. A Superdex GPC column (200 10/300 GL, GE healthcare, Hatfield, UK) was used as the stationary phase and phosphate-buffered saline (50 mM; pH 7.4; 150 mM NaCl) was used as the mobile phase. The flow rate was set up at 0.5 mL/min; polyethylene glycol (PEG) was used as the standard.

### 2.4. Preparation of PLGA and PLGA/PBMP Microparticles

PLGA and PLGA/PBMP microparticles encapsulating Dex were prepared by a previously reported oil-in-water (*o*/*w*) single emulsion/solvent evaporation method with slight modification [16,22]. The organic phase was prepared by mixing a PLGA solution in DCM at a concentration of 5.0% (*w*/*v*) and a Dex solution in acetone at a concentration of 0.5% (*w*/*v*). The mixture was added to a 2.0% (*w*/*v*) PVA solution in water and the total mixture was homogenized at 3000 rpm for 1 min. The resulting emulsion was stirred for 5 h at 30 °C with a magnetic stirrer to evaporate DCM. 10 mL of either deionized water or 1.0% (*w*/*v*) PBMP solution in water was added to prepare Dex-loaded PLGA microparticles (Dex-PLGA), or Dex-loaded PLGA/PBMP microparticles (Dex-PLGA/PBMP), respectively; the mixture was stirred for another 1 h. The particle suspension was centrifuged (Supra 21K; Hanil, Daejeon, Korea) at 15,000 rpm for 10 min and the precipitate was resuspended in deionized water. The washing step was repeated three times. Finally, the particles were obtained by lyophilization. Cy5.5-loaded microparticles were also fabricated by the same procedure using 20 μg of Cy5.5 instead of Dex. 

### 2.5. Scanning Electron Microscopy (SEM)

The fabricated particles were coated with Pt by a sputter (EM ACE200; Leica, Wetzlar, Germany). Then, the morphologies of the particles were examined by field-emission scanning electron microscopy (FE-SEM) (SIGMA; Carl Zeiss, Jena, Germany).

### 2.6. Zeta Potential

The microparticles were suspended in deionized water (3 mg/mL). The surface charge of the particles in the suspension was analyzed by a Zetasizer (Zetasizernano ZS; Malvern, UK).

### 2.7. Measurement of the Dex Loading Amount

The loaded amount of Dex in the microparticles were quantified by high-performance liquid chromatography (HPLC) system (Shimadzu, Kyoto, Japan). For the preparation of the sample, the Dex-loaded particles were dissolved in acetone (1.0 mg/mL). Then, the solution was filtered through a 0.2 μm PVDF membrane filter. The filtered solution was injected to the HPLC system. A C18 column (Eclipse XDB-C18; Agilent, Santa Clara, CA, USA; pore size 5 μm, 4.6 × 250 mm) was used as the stationary phase and a mixture of ACN and water was used as the mobile phase. The flow rate was set up at 1.0 mL/min and the analytes were detected by the UV absorbance at 240 nm (SPD-20A; Shimadzu, Kyoto, Japan).

$$\mathrm{Drug}\;\mathrm{content}\;\left(\mu g/\mathrm{mg}\right)=\frac{\mathrm{mass}\;\mathrm{of}\;\mathrm{drug}\;\mathrm{loaded}\;\mathrm{in}\;\mathrm{particles}}{\mathrm{mass}\;\mathrm{of}\;\mathrm{particles}}$$

$$\mathrm{Encapsulation}\;\mathrm{efficiency}\;\left(\%\right)=\frac{\mathrm{mass}\;\mathrm{of}\;\mathrm{drug}\;\mathrm{loaded}\;\mathrm{in}\;\mathrm{particles}}{\mathrm{mass}\;\mathrm{of}\;\mathrm{drug}\;\mathrm{in}\;\mathrm{the}\;\mathrm{preparation}\;\mathrm{solution}}\times 100$$

### 2.8. HA Binding Affinity Measurement

The binding affinity of PLGA and PLGA/PBMP particles on hydroxyapatite (HA) discs was compared by measuring the fluorescence intensity of the HA disc surface after the treatment of Cy5.5-loaded particles. Briefly, 5 mg of Cy5.5-loaded PLGA or PLGA/PBMP particles were dispersed in 1 mL of deionized water. HA disc was immersed in each particle suspension for 10 min with gentle shaking at ambient temperature. Then, HA discs were washed with fresh deionized water, and sonicated in a bath-type sonicator for 1 min to remove weakly bound particles from the HA surface. The washed HA discs were dried in vacuum at 37 °C overnight. Confocal laser scanning microscopic (CLSM) images of the HA discs were obtained with LSM 880 (Zeiss, Jena, Germany). The fluorescence intensity at excitation and emission wavelength of 633 and 656–758 nm, respectively, were measured using the ImageJ program (NIH, Bethesda, MD, USA). 

### 2.9. In Vitro Drug Release of Dex-Loaded PLGA/PBMP

The Dex-loaded PLGA/PBMP microparticles were suspended in sodium phosphate buffered saline at a concentration of 1 mg/mL. The suspension was incubated with a gentle shaking at 37 °C. At each time point, the suspension was centrifuged at 14,000 rpm for 10 min to separate the particles from the released Dex. An aliquot of the supernatant was carefully collected; the amount of Dex in the aliquot was measured by HPLC.

### 2.10. Establishment of Neo-Osteogenesis Mouse Model

BALB/C mice with 6 weeks of age were used in our study. All 35 mice were anesthetized by inhalation of 2.5% isoflurane (Forane solution Choongwae, Korea) via a vaporizer. The mice were randomly enrolled into five groups (*n* = 7 for each): (A) PBS only, (B) PLGA/PBMP, (C) Dex-PLGA/PBMP, and (D) free Dex. The mechanical injury was induced in the left nasal cavity by insertion of an interdental brush with a radius of 0.6 mm and a clockwise rotation of the brush by five times. The right nasal cavity was preserved to keep breathing. The head was placed lower to the body for 5 min after the mechanical injury to prevent blood aspiration. After 2 h, a regimen including (A) PBS only, (B) PLGA/PBMP, (C) Dex-PLGA/PBMP, and (D) free Dex was administrated intranasally according to each group under the head-fixed conditions. The nasal cavity was irrigated with normal saline before and after intranasal regimen administration. The dose of Dex was 1 mg/kg in (C) and (D). At the time point of day 14 after the sample treatment, the mice were euthanized and decapitated. The procedure was summarized in Figure 5a. The study protocol was approved by the Institutional Animal Care and Use Committee of SMG-SNU Boramae Medical Center on 22 March 2020 (approval no. 2020-0045).

### 2.11. Histological Analysis

The heads of mice were dissected, fixed in 4% (*v*/*v*) paraformaldehyde, and decalcified in the rapid decalcifying solution at 4 °C for 2 days. Then, the tissues were dehydrated and embedded in paraffin by the standard protocol. After Hematoxylin and Eosin (H&E) staining, the samples were analyzed for the evaluation of neo-osteogenesis in the section images, including mucosal detachment, woven bone formation, exudate, and synechia. Particularly, the area of new bone formation, as shown in Figure 5b,c, was estimated by the ImageJ software.

After immunohistochemical staining of osteocalcin, the osteoblast activity in neo-osteogenesis can be estimated. First, the endogenous peroxidase activity in the sample was quenched with 3% of hydrogen peroxide. Then, the epitope was retrieved by using microwave. The samples were treated with a primary antibody (mouse anti-human osteocalcin; 1:100; Santa Cruz Biotechnology; Dallas, TX, USA) for 60 min at room temperature. Finally, the samples were stained with the DAB-Detection (Golden Bridge International Labs, Bothell, WA, USA) and counter-stained with hematoxylin. The numbers of the positive cells in the epithelium and submucosa were counted in the five densest visual fields (×400) by two independent examiners. When the two examiners disagreed with each other, a consensus was reached after review of the specimen under a multi-head microscope by our research team. 

## 3. Results

### 3.1. Synthesis of PBMP

The PBMP was polymerized from DMOEP and BMA monomers by free radical polymerization according to a previously reported procedure [16,21]. The monomer content in the synthesized polymer was analyzed by ^1^H NMR (Figure S1a, Supporting Information), showing 46% MOEP and 54% BMA in the polymer. The molecular weight distribution was analyzed by GPC. The number average molecular weight (*M*_n_) and the weight average molecular weight (*M*_w_) of PBMP were 16,900 g/mol and 32,200 g/mol, respectively (Figure S1b, Supplementary Materials). The polydispersity (PD) index of 1.91 was a typical characteristic of polymers synthesized by free radical polymerization.

### 3.2. Preparation and Characterization of Dex-Loaded PLGA/PBMP Microparticles

**Figure 2:**
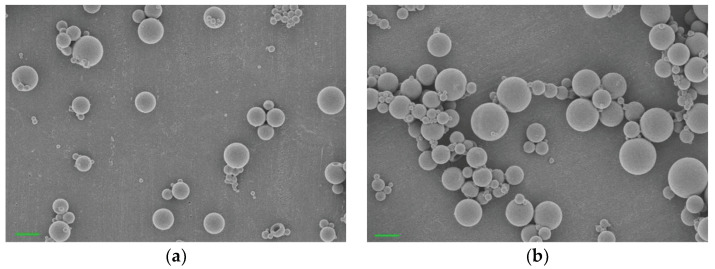
The representative FE-SEM images of Dex-loaded (**a**) PLGA microparticles and (**b**) PLGA/PBMP microparticles. Each scale bar represents 1 μm.

A single oil-in-water emulsion/evaporation method was used for the encapsulation of Dex into PLGA microparticles. After encapsulation, PBMP could be coated on the surface of the PLGA particles by simple immersion. The morphologies of the PLGA and PLGA/PBMP particles are shown in the FE-SEM images (Figure 2a,b). Both particles showed spherical shapes with average diameters of 0.4–1.0 μm. The more negative zeta potential of the PLGA/PBMP microparticles (−44 mV) than that of the non-coated PLGA microparticles (−26 mV) confirmed the successful introduction of the negatively charged phosphomonoester group of PBMP on the surface of the microparticles (Table 1) [16].

The amount of Dex loaded in the particles was analyzed using HPLC. As shown in Table 1, Dex-PLGA and Dex-PLGA/PBMP showed similar drug contents (12.85 μg/mg and 12.79 μg/mg, respectively) and encapsulation efficiency (10.28%, and 10.23%, respectively). The similar values between Dex-PLGA and Dex-PLGA/PBMP imply that the PBMP coating procedure did not significantly affect the encapsulation of Dex into the microparticles. 

### 3.3. HA Binding Affinity of PLGA/PBMP Microparticles

**Figure 3:**
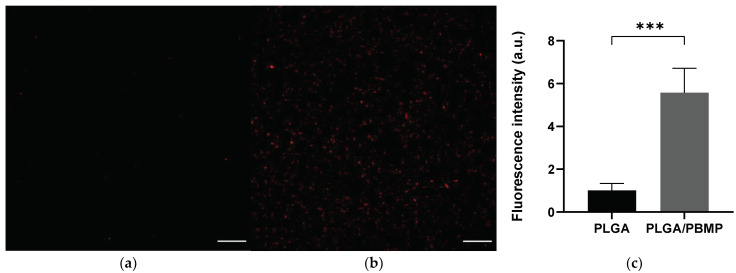
Enhancement of the HA-binding affinity of PLGA particles by PBMP coating. The representative CLSM images of the HA discs after incubation with Cy5.5-loaded (**a**) PLGA or (**b**) PLGA/PBMP particles, respectively. Red fluorescence indicates the position of particles. Each scale bar represents 100 μm. (**c**) Comparison of fluorescence intensities in the CLSM images. The fluorescence intensity was measured in randomly selected areas of each HA disc (*n* = 12). The bar represents the mean ± SEM. The statistical analysis was performed using Student’s unpaired *t*-test. *** indicates *p* < 0.001.

The HA binding affinity of PLGA and PLGA/PBMP microparticles was compared using fluorescence-dye (Cy5.5)-encapsulated particles. HA discs were immersed in either Cy5.5-PLGA or Cy5.5-PLGA/PBMP suspension and thoroughly washed with fresh water with sonication to remove weakly bound particles from the surface. As presented in the CLSM images (Figure 3), the HA disc incubated in the Cy5.5-PLGA/PBMP suspension showed 5.6-times higher fluorescence intensity than the disc incubated in the Cy5.5-PLGA suspension. The PBMP coating greatly enhanced the HA-binding affinity of PLGA particles.

### 3.4. In Vitro Drug Release of Dex-Loaded PLGA/PBMP Microparticles

In vitro release of Dex from the PLGA/PBMP microparticles was examined by HPLC (Figure S2, Supporting Information). There is an initial burst up to 38% within 1 h. Then, the release was slowed down with the cumulative release of ~50% for 1 day. The Dex release continued at a slower rate. At the time point of the 7th day, the cumulative Dex release was approximately 70%. The sustained release of Dex was maintained over 7 days in the PLGA/PBMP microparticle system. 

### 3.5. In Vivo Effect of Dex-Loaded PLGA/PBMP Microparticles

**Figure 4:**
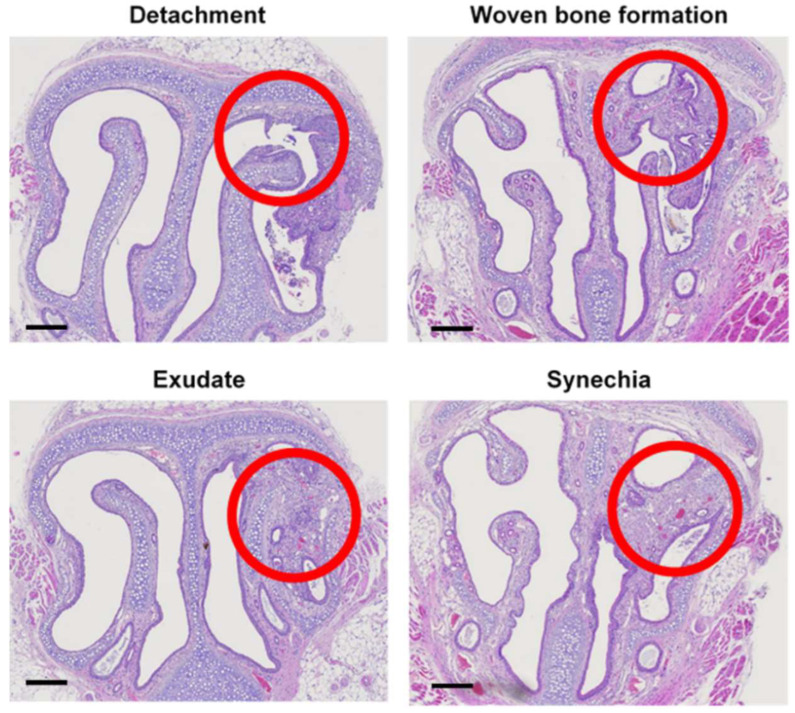
Representative H&E-stained images of sinonasal cavity treated after the mechanical injury of mucosa in mice. The mucosal detachment, woven bone formation, exudate and synechia were indicated as circles. The scale bar represents 500 μm.

The H&E histologic features of the mechanically injured side of all mice (left) showed mucosal detachment, woven bone formation, exudate, and synechia around the mouse nasoturbinate bone (Figure 4). These histological findings are typical characteristics of neo-osteogenesis, indicating that the neo-osteogenesis mouse model was well established. 

**Figure 5:**
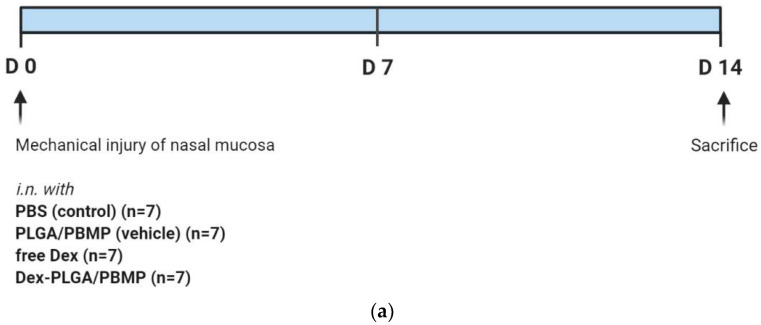

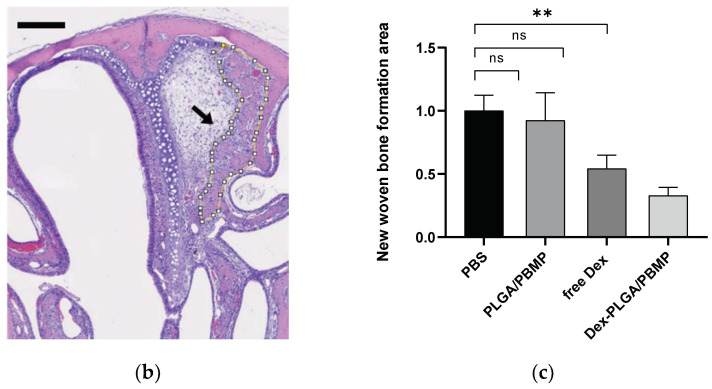
(**a**) Schematic illustration of the in vivo study protocol: Dex (Dexamethasone); PLGA/PBMP (empty PLGA/PBMP particles); Dex-PLGA/PBMP (Dex-loaded PLGA/PBMP particles); *i.n.* (intranasal). (**b**) A representative H&E-stained image for the estimation of new bone formation area (dotted yellow line) in the sinonasal cavity of the mouse model treated with PBS only. The scale bar represents 500 μm. (**c**) Relative area of woven bone formation. The bar indicates the mean ± SEM of the area of woven bond formation (*n* = 7). The statistical analysis was performed using one-way ANOVA test. ** indicates *p* < 0.01 and ns means not significant.

The cross-sectional area of new bone formation was measured in the H&E-stained images to compare the neo-osteogenesis alleviation in each group (Figure 5b). The mice treated with free Dex and Dex-loaded PLGA/PBMP showed a notable reduction in new bone formation compared to the PBS control. The free PLGA/PBMP showed no remarkable difference from the PBS control. The relative area of the new woven bone formation was quantitatively compared among the groups. Only Dex-loaded PLGA/PBMP showed a statistically significant reduction in woven bone formation compared to the PBS control. Free Dex showed some, but no significant, reduction in bone formation. The effect of free PLGA/PBMP on bone formation was negligible (Figure 5c).

**Figure 6:**
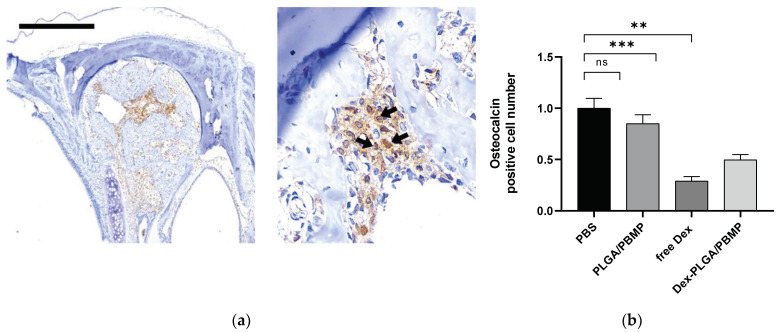
Comparison of osteocalcin activity in the IHC images of the sinonasal cavity treated with various formulations. (**a**) Representative antibody-stained images image showing the osteoblast-related bone regeneration in the mucosal defect area. The black arrows show the osteocalcin-positive cells. The scale bar represents 500 μm. (**b**) Relative osteocalcin activity. The relative numbers of osteocalcin-positive cells were compared. The bar indicates the mean ± SEM (*n* = 7). The statistical analysis was performed using one-way ANOVA test. ** and *** indicate *p* < 0.01 and *p* < 0.001, respectively. ns means not significant.

We compared the osteocalcin activity of the nasal septum and the nasoturbinate area using immunohistochemistry (IHC). The relative numbers of osteocalcin-positive cells are compared in Figure 6. Similar to the results of the new woven bone formation, free Dex and Dex-loaded PLGA/PBMP showed approximately 30–50% of osteocalcin activity compared to the PBS control. However, unlike the bone formation results, free Dex showed the lowest osteocalcin activity, followed by Dex-loaded PLGA/PBMP.

## 4. Discussion

CRS is considered to be caused by multifactorial factors such as genetic, environmental, and infectious causes in the paranasal sinus [23]. Although the medical use of antibiotics or steroid drugs is clinically preferred against CRS, some patients show limited responses to non-surgical treatment. Surgical treatment should be selected for such patients [24]. However, many patients with abnormal findings show poor prognostic outcomes even after ESS [10,11]. It has been reported that recalcitrant CRS is closely related to nasal polyps, adhesion, synechiae formation, ostial stenosis, and bony osteitis [24].

The pathogenesis of bony changes after surgery is not fully understood and is a relatively new clinical entity. Chronic inflammation, mechanical stress, and post-traumatic repair may initiate bone remodeling, including periosteal reactions, osteoclast proliferation, bone resorption, new bone formation, and fibrosis [8,12]. Interestingly, the incidence of osteitic bones increases with an increasing number of previous operations [25], which implies that mucosal injury secondary to surgery is responsible for the development of osteitis. Some degrees of mucosal tearing and detachment, as well as bony exposure, are sometimes unavoidable even by careful ESS procedure to preserve the mucosa. Infection or inflammation around the mucosal damage may initiate osteitis [12]; chronic inflammation in the bone may, conversely, inhibit the effective regeneration of the mucosa and induce the recurrence of CRS [4]. Therefore, if we could successfully inhibit surgery-induced osteitis, we could also expect to enhance mucosal regeneration and to reduce the recurrence ratio of CRS.

The treatment of CRS-related osteitis remains controversial [26,27]. Several studies have advocated the improvement of radical surgery to remove all involved bones [7,28], but recent studies have shown worse outcomes after radical surgery [11]. In addition, macrolide antibiotics inhibit osteitis or bone remodeling in vitro [29], but it is difficult to use antibiotics for the treatment of osteitis for a long duration. A postoperative corticosteroid regimen may be necessary for patients with serious inflammation [8]. Steroid treatment could effectively moderate sinus inflammation, which is crucial for the initiation of osteitis in the adjacent bones [30]. It also has a strong effect in facilitating the wound healing process after surgery, along with reduction of subepithelial edema and adhesion formation [14].

However, there are always serious safety concerns regarding the systemic use of corticosteroids, including sleep disturbance, psychological effects, metabolic disorders, peptic ulceration, heart failure, and avascular necrosis [15]. Even in the systemic treatment of corticoids after ESS, delayed mucosal ciliary regeneration was observed in the rat model [14]. Therefore, topical delivery of corticoids is often preferred to avoid the adverse effects of systemic delivery, and various modalities such as nasal spray or irrigation have been developed. However, local corticosteroids can be rapidly diffused or washed out; eventually, it is difficult to maintain the effective concentration of corticoids that is limited to the target area, i.e., in this study, the wounded area and adjacent bones. 

In this study, we envisioned that the local release of corticoids only around the wounded area and exposed bones using PBMP-coated PLGA microparticles would effectively prevent the development of osteitis with minimal side effects. The PBMP-coated PLGA particles could bind the exposed bone after the surgery and slowly release the encapsulated corticoids to maintain the effective concentration in the tissues only around the particles adhered to the bone (Figure 1).

In our previous report, a calcium-binding polymer, PBMP, was designed to enhance the binding affinity of PLGA particles to HA [16]. To simultaneously interact with both the calcium-rich bone surface and hydrophobic PLGA particle surface, the MOEP monomer with a calcium-binding phosphomonoester group and the BMA monomer with a hydrophobic alkyl group were incorporated into the PBMP polymer. PBMP was successfully synthesized via free radical polymerization (Figure S1). The possible cytotoxicity of PBMP was examined in a previous report using pre-osteoblasts (MC3T3-E1), showing no significant cytotoxicity up to a concentration of 1000 μg/mL [16].

We selected Dex as a model corticoid drug because it was reported to repress the expression of many inflammatory cytokines and has a therapeutic effect on the wound healing process after the surgical procedure [6,14,20]. To encapsulate Dex into the PLGA microparticles, we used a single oil-in-water emulsion/evaporation method [16,22]. Then, without requiring complicated conjugation chemistry, the calcium binding ability of the PBMP-coated PLGA particles could be obtained by simple immersion of PLGA particles into the aqueous solution of PBMP. As shown in the FE-SEM images (Figure 2), both Dex-PLGA and Dex-PLGA/PBMP showed spherical shapes with an average diameter of approximately 0.7 μm. Compared to the sizes of the particles (40–50 μm) in our previous report [18], we obtained much smaller-sized microparticles by using strong homogenization of the oil-in-water emulsion. It was expected that the smaller size can guarantee the larger number of phosphomonoester groups/mass of the particles and may provide stronger calcium binding property enough to stabilize the bound PLGA/PBMP around the exposed bones in the nasal cavity with severe mucociliary activity. Along with similar Dex content (13 μg/mg) and encapsulation efficiency (~10%) of Dex-PLGA and Dex-PLGA/PBMP, we could infer that the PBMP coating had no significant influence on the morphology of the PLGA particles and the Dex encapsulation. The zeta potential of Dex-PLGA/PBMP was different from that of Dex-PLGA. The significantly lower zeta potential value of Dex-PLGA/PBMP than that of Dex-PLGA clearly indicated that PBMP was successfully coated on the PLGA particle surface to expose the negatively charged phosphomonoester groups. 

Although terminal carboxylates on the surface of bare PLGA particles can weakly interact with the calcium ions of bones, a stronger binding affinity might be preferred to prevent the detachment of the particles from the exposed nasal bone by body fluids. To assess the bone-binding affinity of PLGA and PLGA/PBMP microparticles, we compared the number of particles bound on discs of HA, the main component of natural bones, using fluorescent dye (Cy5.5)-loaded microparticles; more than 5-times higher fluorescence intensity was observed even after thorough washing and sonication (Figure 3), which confirmed the enhanced HA-binding property of the PLGA particles by PBMP coating. It is assumed that the high density of divalent phosphomonoester groups on the PLGA/PBMP particle surface provided far stronger interactions with HA than the monovalent terminal carboxylates on the PLGA particles [31,32]. We expected that the PBMP-coated PLGA particles could adhere to the surface of the exposed bones after the surgery with sufficient stability to release the encapsulated Dex for a long duration. 

We could observe an initial burst and the successive sustained release of Dex from the PLGA/PBMP particles (Figure S2). The initial burst was originated from encapsulated Dex near the surface of the particle, which would rapidly diffuse out as water penetrates into the polymer shell. Then, the particle swelling and PLGA hydrolysis would further facilitate the release of Dex in the inner part of the particle during next several days [33,34]. Since the results supported that the sustained Dex release was maintained over 7 days, we expect that the Dex-PLGA/PBMP particles can maintain the Dex concentration nearby the bound area for a longer period than the free Dex does, which rapidly diffuses throughout the nasal cavity and becomes washed out.

We then applied the Dex-loaded PLGA/PBMP particles to the neo-osteogenesis model in the mouse nasal cavity. As shown in Figure 4, the mechanical injury of the mouse nasal mucosa using an interdental brush could induce synechia, exudate, fibrosis, osteoblastic activity with woven bone formation, and the characteristics of neo-osteogenesis in the tissue near the injured position. The postoperative neo-osteogenesis model was established using this method. 

Intranasal administration of Dex-PLGA/PBMP particles after nasal mucosal injury showed beneficial effects during the wound healing process. Only Dex-PLGA/PBMP particles showed statistically significant inhibition (~70%) of the new woven bone formation compared to the PBS control (Figure 5). Intranasal administration of free Dex resulted in an approximately 50% reduction in the woven bone formation on average, but the difference was not statistically significant due to the large standard deviation. The sustained release from the PLGA/PBMP particles might maintain the Dex concentration around the exposed bone area for a longer period than free Dex to inhibit postoperative bone remodeling more effectively. 

The IHC results for the measurement of osteocalcin activity also showed a statistically significant beneficial effect of Dex-PLGA/PBMP particles compared to the PBS control (Figure 6). However, in osteocalcin activity, Dex-PLGA/PBMP showed a slightly higher average value than free Dex, although the difference was not statistically significant. Since osteocalcin is mainly expressed in the late stage of bone remodeling, we should further investigate the time-dependent relationship between osteocalcin expression and woven bone formation in future studies.

Our study suggests a promising opportunity for the clinical application of local Dex delivery systems targeted to exposed bone in the field of recalcitrant CRS-related sinus surgery by using calcium-binding biodegradable polymeric vehicles. However, this study had certain limitations. As for the materials, the Dex release kinetics should be further optimized for better efficacy for the treatment of postoperative osteitis. Although it is known that topical steroid administration gives significant effects in the early postoperative period, as it can minimize the mucosa inflammation, synechia and other complications, and enhances overall wound healing process [20], there is only limited information about the most effective concentration and duration of the treatment. Since release kinetics are highly dependent on the ratio between lactic acid and glycolic acid in PLGA as well as the pH conditions [33], we could finely tune the release profile considering the preferred maintenance of the Dex concentration in the nasal inflammatory environment. We could better understand the relationship between the Dex releasing kinetics and neo-osteogenesis by treating PLGA/PBMP particles with different release kinetics on pre-osteoblasts. In the animal model, there may be differences in the wound healing process between the mucosa of CRS patients after ESS and the mucosa of mice that were artificially injured by the brush. Additionally, a larger animal model such as a rabbit, which has a nasal cavity and metabolic rates that are closer to those of humans than mice, might be preferred to examine the specific binding of Dex-PLGA/PBMP on the exposed bone and the wound healing process related to postoperative osteitis. More detailed biochemical analyses other than the osteocalcin assay are required to elucidate the inhibitory mechanism of Dex on bone remodeling. Furthermore, owing to the encapsulating capability of PLGA microparticles, a versatile combination of corticoids and other drugs could be applied to the postoperative neo-osteogenesis model for better efficacy without minimal side effects. Importantly, the safety of the Dex-PLGA/PBMP formulation for intranasal administration should be carefully investigated in future clinical applications.

## 5. Conclusions

In summary, we demonstrated a calcium-binding drug delivery system, PLGA/PBMP, for the sustained delivery of drugs around the exposed bone materials and investigated its effectiveness in the delivery of corticoids to the wounded tissue around the exposed nasal bone after the surgical injury. The PBMP coating strongly enhanced the binding affinity of Dex-loaded PLGA microparticles onto the calcium-rich surface. When intranasally administered to the sinonasal cavity of the surgery-induced neo-osteogenesis mouse model, Dex-PLGA/PBMP showed a significant inhibitory effect on new woven bone formation and osteocalcin expression in the wounded area, implying its potential to inhibit the development of osteitis and enhance postoperative wound recovery. Although further studies are required to optimize the efficacy of larger osteitis models, the aforementioned results demonstrated that the PLGA/PBMP system is a promising drug delivery strategy for the effective treatment of CRS-related osteitis.

## Figures and Tables

**Table 1:** Characteristics of Dex-loaded PLGA and PLGA/PBMP microparticles ^a^.

Microparticles	Size (μm) ^b^	Zeta Potential (mV) ^c^	Dex Loading ^c^	
			Drug Content (μg/mg)	Encapsulation Efficiency (%)
Dex-PLGA	0.69 ± 0.28	−26.62 ± 0.80	12.85 ± 0.89	10.28 ± 0.71
Dex-PLGA/PBMP	0.68 ± 0.33	−44.01 ± 0.80	12.79 ± 0.02	10.23 ± 0.02

^a^ Each value is represented as the mean ± standard deviation (S.D.). ^b^ The particle size was measured in FE-SEM images (*n* > 1000). ^c^ The other values were obtained from three samples (*n* = 3).

## Data Availability

No new data were created or analyzed in this study.

## Supplementary Materials

The following supporting information can be downloaded at: www.mdpi.com/article/10.3390/pharmaceutics14030546/s1, Figure S1: Synthesis of poly(butyl methacrylate-*co*-methacryloyloxyethyl phosphate) (PBMP); Figure S2: In vitro release profile of Dex-PLGA/PBMP microparticles.
