# Supplementary material for: Alleviation of Surgery-Induced Osteitis in Sinonasal Cavity by Dexamethasone-Loaded Poly(lactic-co-glycolic acid) (PLGA) Microparticles with Strong Calcium-Binding Affinity
Source: Pharmaceutics. 2022 Feb 28;14(3):546. doi: 10.3390/pharmaceutics14030546 (PMC8950508; doi:10.3390/pharmaceutics14030546)
Supplement: Supplementary file 1 [file pharmaceutics-14-00546-s001.zip › pharmaceutics-1568057-supplementary.pdf]

pharmaceutics

Supplementary Materials: Alleviation of Surgery-Induced Osteitis in Sinonasal Cavity by Dexamethasone-Loaded Poly(lactic-*co*-glycolic acid) (PLGA) Microparticles with Strong Calcium-Binding Affinity

(a)

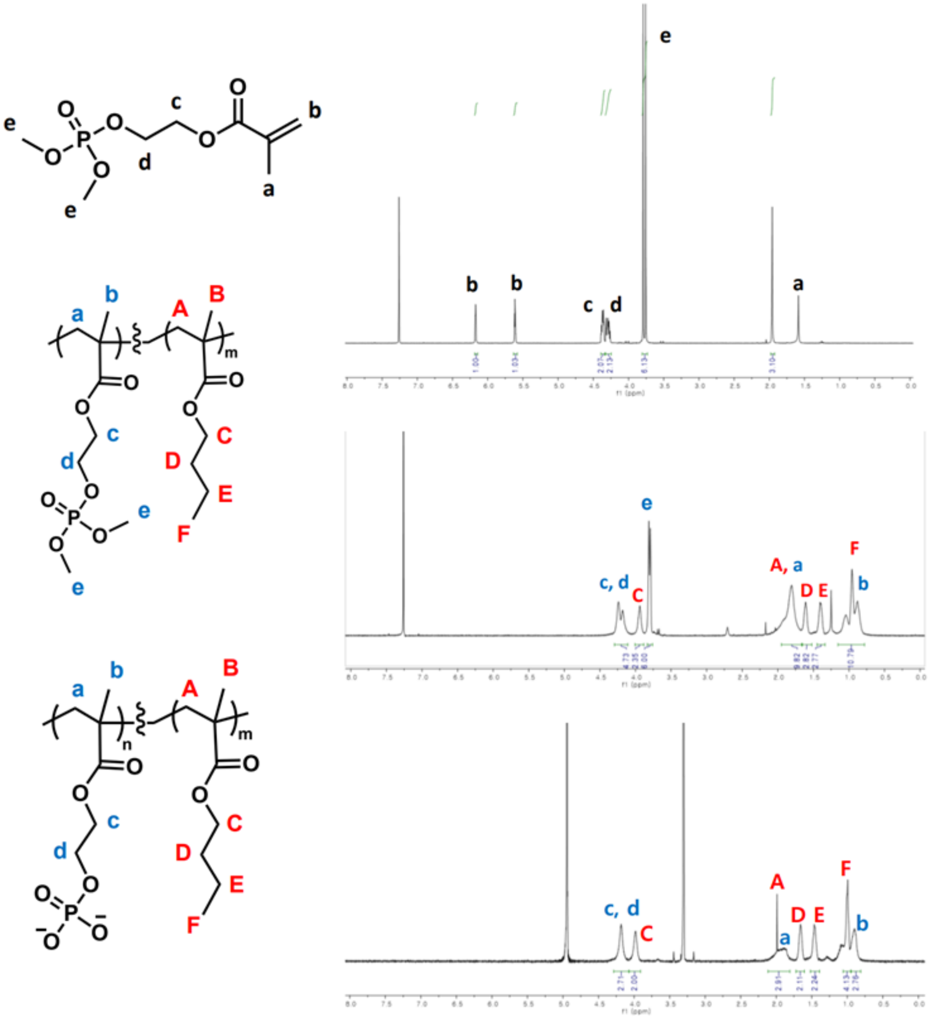

(b)

MOEP composition (mol%)[a]		Averaged molecular weight[b]		PD (M_w/M_n)
Feed	In polymer	M_n	M_w	
50.0	45.9	1.69 x 10^4	3.22 x 10^4	1.91

[a] Determined by ^{1}H NMR.

[b] Determined by GPC using PEG standards.

Figure S1. Synthesis of poly(butyl methacrylate-*co*-methacryloyloxyethyl phosphate) (PBMP). (a) ^{1}H-NMR spectra of DMOEP in $CDCl_3$, poly(BMA-*co*-DMOEP) in $CDCl_3$, and PBMP in CD_3OD, respectively. (b)The composition and molecular weight information of PBMP.

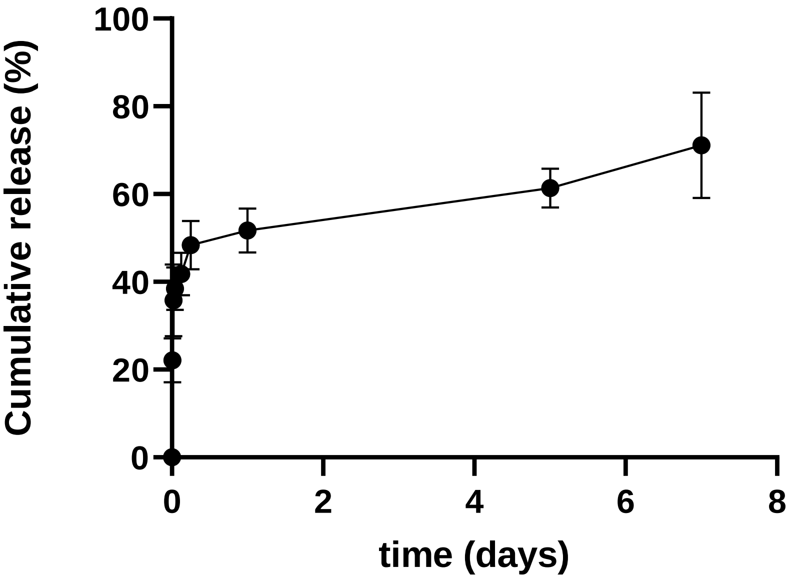

Figure S2. *In vitro* release profile of Dex-PLGA/PBMP microparticles in PBS medium at 37 °C. Each data point represents average ± S.D. (n = 5).
